# Factors Associated with Abortion Complications after the Implementation of a Surveillance Network (MUSA Network) in a University Hospital

**DOI:** 10.1055/s-0041-1735129

**Published:** 2021-08-30

**Authors:** Camila Ayume Amano Cavalari, Nelio Neves Veiga-Junior, Beatriz Deguti Kajiura, Caroline Eugeni, Barbara Virgínia Gonçalves Tavares, Luiz Francisco Baccaro

**Affiliations:** 1 Department of Obstetrics and Gynecology, Faculty of Medical Sciences, State University of Campinas – UNICAMP – Brazil

**Keywords:** CLAP MUSA network, perinatal information system, population surveillance, abortion, EviSIP, rede CLAP MUSA, sistema de informação perinatal, vigilância populacional, aborto, EviSIP

## Abstract

**Objective** To evaluate the factors associated with abortion complications following the implementation of the good-practice surveillance network Mujeres en Situación de Aborto (Women Undergoing Abortion, MUSA, in Spanish).

**Methods** A cross-sectional study with women who underwent abortion due to any cause and in any age group at UNICAMP Women's Hospital (part of MUSA network), Campinas, Brazil, between July 2017 and Agust 2019. The dependent variable was the presence of any abortion-related complications during hospitalization. The independent variables were clinical and sociodemographic data. The Chi-square test, the Mann–Whitney test, and multiple logistic regression were used for the statistical analysis.

**Results** Overall, 305 women were enrolled (mean ± standard deviation [SD] for age: 29.79 ± 7.54 years). The mean gestational age was 11.17 (±3.63) weeks. Accidental pregnancy occurred in 196 (64.5%) cases, 91 (29.8%) due to contraception failure. At least 1 complication was observed in 23 (7.54%) women, and 8 (34.8%) of them had more than 1. The most frequent complications were excessive bleeding and infection. The factors independently associated with a higher prevalence of complications were higher gestational ages (odds ratio [OR]: 1.22; 95% confidence interval [95%CI]: 1.09 to 1.37) and contraceptive failure (OR: 3.4; 95%CI: 1.32 to 8.71).

**Conclusion** Higher gestational age and contraceptive failure were associated with a higher prevalence of complications. This information obtained through the surveillance network can be used to improve care, particularly in women more susceptible to unfavorable outcomes.

## Resumo

**Objetivo** Avaliar os fatores associados às complicações em casos de aborto após a implementação da rede de vigilância de boas práticas Mujeres en Situación de Aborto (Mulheres em Situação de Aborto, MUSA, em espanhol).

**Métodos** Um estudo transversal, com mulheres admitidas por aborto de qualquer causa e em qualquer faixa etária, no Hospital da Mulher da UNICAMP (parte da rede MUSA), Campinas, Brasil, entre julho de 2017 e agosto de 2019. A variável dependente foi a presença de qualquer complicação relacionada ao quadro de aborto durante a hospitalização. As variáveis independentes foram dados clínicos e sociodemográficos. O teste de qui-quadrado, o teste de Mann-Whitney, e a regressão logística múltipla foram usados na análise estatística.

**Resultados** Foram incluídas 305 mulheres (média ± desvio padrão [DP] da idade: 29,79 ± 7,54 anos). A idade gestacional média foi de 11,17 (±3,63) semanas. A gravidez não foi planejada em 196 (64,5%) casos, 91 (29,8%) devido a falha de contraceptivo. Pelo menos 1 complicação foi observada em 23 (7,64%) mulheres, 8 (34,8%) das quais apresentaram mais de uma complicação. As complicações mais frequentes foram sangramento excessivo e infecção. Os fatores independentemente associados à maior prevalência de complicações foram idades gestacionais maiores (razão de chances [OR]: 1.22; intervalo de confiança de 95% [IC95%]: 1.09 a 1.37) e falha de contraceptivo (OR: 3.4; IC95%: 1.32 a 8.71).

**Conclusão** Maior idade gestacional e falha de contraceptivo estiveram associados à maior prevalência de complicações. As informações obtidas pela rede de vigilância podem ser usadas para melhorar o cuidado, particularmente nas mulheres mais suscetíveis a desfechos desfavoráveis.

## Introduction

Bleeding is a common complication in the first trimester of pregnancy. It is estimated that ∼ 12% to 24% of women with a missed menstrual period and a positive pregnancy test do not progress to full-term pregnancy. 1 The differential diagnoses of first-trimester bleeding include abortion, ectopic pregnancy, and gestational trophoblastic disease, which, for the most part, have spontaneous etiologies. 1 However, a significant proportion of abortions may be due to unwanted pregnancies and unsafe provoked abortions. Every year, an estimated 121 million unwanted pregnancies occur worldwide, with 61% ending in abortion. 2 The overall complication rate after an unsafe abortion is estimated at 6.9 per 1,000 women aged 15 to 44 years. 3

According to the World Health Organization (WHO), the worldwide prevalence of maternal deaths from abortion is estimated at 7.9%, with the highest percentages found in Sub-Saharan Africa (9.6%) and Latin America and the Caribbean (9.9%). 4 More specifically, in Brazil, despite the reduction in the number of severe abortion-related complications, cases of preventable morbidity and mortality persist. 5 According to Santana et al., 6 2.5% of obstetric complications in Brazil are due to abortion, 81.9% of which because of potentially life-threatening conditions, 15.2% due to maternal near miss, and 3% because of maternal deaths. 6

It is challenging to study the quality of obstetric care in women undergoing abortion in countries with restrictive abortion laws and associated ethical and moral dilemmas, clandestine abortion, fear of social stigma, and prosecution. 7 Thus, we aimed to evaluate the factors associated with abortion complications after the implementation of a good-practice surveillance network in a university hospital in the southeastern region of Brazil.

## Methods

We performed a cross-sectional prospective study from July 1st, 2017, to August 31, 2019. We included women admitted to our institution who underwent abortion due to any cause and from any age group. Moreover, we excluded cases of bleeding during pregnancy due to causes other than abortion. The present study was approved by the Ethics in Research Committee of our institution (under CAAE 62778316.6.1001.5404).

### Data Collection

Mujeres en Situación de Aborto (Women Undergoing Abortion, MUSA, in Spanish) is a multicenter network with international cooperation created by Centro Latinoamericano de Perinatología (Latin American Center for Perinatology, CLAP, in Spanish) to encourage good practices in the care of women during abortions in Latin America and the Caribbean. 8 9

This network compiles data related to the pregnancy-puerperal cycle from several hospitals in many countries in Latin America through Sistema de Información Perinatal (Perinatal Information System, SIP, in Spanish), a software developed by CLAP to facilitate the recording of this data. Each sentinel center is responsible for regularly feeding the database with information such as maternal morbidity and maternal near miss in early gestational loss, method of choice for uterine evacuation ratio, prevalence of complications related to the termination of early pregnancy, preoperative antibiotic prophylaxis ratio, contraceptive counselling, and initiation prior to hospital discharge. In addition, it enables epidemiological monitoring and the comparison of different sentinel centers over time. There are also regular online meetings with delegates from each sentinel center to discuss the data collected, and scientific discussions on the subject of care for women in abortion. In the present study, data were obtained through a specific section of the SIP for cases of gestational loss in the first half of pregnancy.

Our institution is a tertiary-level hospital located in the southeastern region of Brazil, which receives cases of pregnancy-related complications from several cities in the region. The hospital has been one of the sentinel centers of the MUSA Network in Brazil since July 2017, and it manages an average of 250 deliveries and 20 first-trimester pregnancy complications monthly. Moreover, the hospital follows the country's laws regarding the possibility of legal termination of pregnancy, that is, in cases of sexual violence, risk of maternal death, and fetal anencephaly. All women who agreed to participate in the project signed a free and informed consent form. Minors signed the assent form, whereas the consent form was signed by their legal guardians.

### EviSIP

This article was written using a new SIP-based method of generating information, called Evidencias del SIP (Evidence from SIP, EviSIP, in Spanish), which has been previously described in detail. 9 In summary, junior researchers from sentinel centers participating in the MUSA Network meet with specialists in human reproduction research from other parts of the world. After the detailed analysis of data from each sentinel center, the most interesting results are submitted for publication in scientific journals. This initiative develops the research ability of professionals in sentinel centers and increases knowledge about reproductive health in Latin America and the Caribbean. 9

### Dependent Variable

The dependent variable was the presence of any abortion-related complication during hospitalization, such as genital infection, pelvic infection, sepsis, excessive bleeding, hypovolemic shock, pelvic organ injury, need for hysterectomy, need for surgical reapproach, presence of intraoperative complications, anesthetic complications, or pregnancy-related clinical complications.

### Independent Variables

The independent variables included: age, literacy, level of schooling, marital status, living alone, morbid medical history (hypertension, tuberculosis, diabetes, preeclampsia, eclampsia, genitourinary diseases, heart disease, infertility), number of pregnancies, number of births, number of abortions, body mass index, active smoking status, drug use, alcohol consumption, planned pregnancy, failure of contraceptive method (CM), date of admission, date of resolution of the last pregnancy, pain score, abortion for legal reasons, gestational age, duration of symptoms, transport time to hospital, surgical uterine evacuation, and use of misoprostol before uterine evacuation.

### Sample Size

The sample size was calculated using the method proposed by Tabachnick and Fidell. 10 Using an α level (type-I error) of 5% (α = 0.05) and a sample power of 80%, the minimum sample size required to observe a significant difference was estimated at 290 women.

### Statistical Analysis

Initially, a descriptive data analysis was performed. The continuous variables are expressed as the mean, standard deviation, median, and minimum and maximum quartiles. The categorical variables are expressed as relative frequencies. For the independent categorical and continuous variables respectively, the Chi-squared or the Fisher's exact and the Mann–Whitney U tests were used to evaluate the factors associated with a higher frequency of abortion-related complications. Multiple logistic regression analysis, with stepwise variable selection criteria, was used to evaluate the factors independently associated with complications. The significance level was set at 5%. The Statistical Analysis System. (SAS, SAS Institute, Inc., Cary, NC, US) software, version 9.2 for Windows, was used for the data analysis.

## Results

From July 2017 to August 2019, 305 women hospitalized in our institution were included in the study, with mean and median ages of 29.79 (±7.54) years and 29 (12 to 48) years respectively. Legal pregnancy termination was performed in 39/305 (12.79%) cases, 34 (87.17%) of which resulted from sexual violence. The mean gestational age was of 11.17 (±3.63) weeks, and 44.27% of the women had been previously pregnant at least twice. At least one previous abortion was observed in 34% of the sample. Accidental pregnancy and pregnancy due to failure of the CM occurred in 196/305 (64.5%) and 91 (29.83%) women respectively. The numbers of women who had a high school degree and had a partner were 255/305 (83.9%) and 195 (65.2%) respectively. Details of the clinical and sociodemographic characteristics are shown in Table 1 .

**Table 1:** Clinical and sociodemographic characteristics of the study sample ( *n* = 305)

Characteristics	n	%
*Diagnosis*		
Spontaneous abortion	266	87.2
Legal abortion	39	12.8
*Age (years)*		
< 20	20	6.6
20–29	138	45.2
30–39	113	37
40–49	34	11.1
*Level of schooling* ^a^		
Primary	48	15.8
Secondary	190	62.5
Higher	65	21.4
*Marital status* ^a^		
Married	122	40.8
Cohabiting	73	24.4
Single	78	26.1
Other	26	8.7
*Previous pregnancy*		
0	97	31.8
1	73	23.9
≥ 2	135	44.3
*Planned pregnancy* ^a^		
Yes	108	35.5
No	196	64.5
* Contraceptive method ^a^*		
None	211	69.9
Hormonal	58	19.2
Barrier	20	6.6
Intrauterine device	7	2.3
Emergency	4	1.3
Natural	2	0.7
*Smoking* ^a^		
Yes	36	11.9
No	266	88.1
*Drug use* ^a^		
Yes	17	5.6
No	284	94.4

Note: ^a^ Data were missing for level of schooling ( *n* = 1), marital status ( *n* = 6), planned pregnancy ( *n* = 1), contraceptive method ( *n* = 4), smoking ( *n* = 4), and drug use ( *n* = 4).

The mean duration of symptoms was of 3.9 (±8.38) days. Misoprostol was administered to 170 (55.74%) women, 28 (16.5%) of whom were not submitted to complementary uterine evacuation. The mean interval time between the administration of misoprostol and uterine evacuation was of 19.62 (±15.6) hours ( Table 2 ). At least 1 complication was observed in 23/305 (7.54%) women, 8 of whom presented with more than 1 complication. The most frequent complication was excessive bleeding in 14 cases, 11 of which required hemoderivative transfusion. Pelvic infection was noted in 11 women, 4 of whom had sepsis. In three cases, a second surgical approach was necessary. The details of complications during the study period are shown in Table 3 .

**Table 2:** Uterine evacuation and methods used ( *n* = 305)

	n	%
*Uterine evacuation*		
Yes	279	91.5
No	26	8.5
*Method used*		
Misoprostol + curettage	152	49.8
Curettage	99	32.4
Misoprostol	28	9.2

**Table 3:** Abortion-related complications ^a^ ( *n* = 23)

	n	Prevalence among women with complications (%)	Prevalence among all women (%)
Excessive bleeding	14	60.8	4.6
Hemoderivative transfusion	11	47.8	3.6
Pelvic infection	11	47.8	3.6
Sepsis	4	17.3	1.3
Surgical reapproach	3	13	0.9
Hysterectomy	3	13	0.9
Uterine perforation	1	4.3	0.3
Disseminated intravascular coagulation	1	4.3	0.3

Note: ^a^ Women could present more than one complication.

Higher rates of adverse events were observed in women who became pregnant after CM failure ( *p* = 0.004) and in those with a higher gestational age ( *p* = 0.002). The final multiple logistic regression model showed a greater risk of developing complications in women with the highest gestational ages (the risk increased by 22.9% each week) and in those with CM failure (3.4-fold risk of complication) ( Table 4 ).

**Table 4:** Factors associated with complications ^a^ ( *n* = 273)

Variables	Categories	*p* -value	Odds ratio	95% confidence interval
Gestational age	Continuous variable (weeks)	**< 0.001**	1.229	1.097–1.378
Contraceptive failure	None (referece level) All the others	— 0.011	1.00 3.4	— 1.32–8.71

Notes: Cases with missing variables were not included in the multiple analysis. ^a^ Multiple logistic regression by stepwise criteria variable selection to determine factors associated with complications (no: *n* = 252; yes: *n* = 21).

## Discussion

The prevalence of complications in women admitted for abortion in the present study was of 7.54%. This rate was higher than that found by Santana et al. 6 in Brazil and in a study 11 conducted in the United States with 54,911 cases of abortion, which were of 2.5% and 2.1% respectively. Our hospital is a teaching institution with resident doctors in training, which may partially explain the higher frequency of complications. 12 Moreover, our hospital is a tertiary-care level hospital, which receives severe cases referred from all over the region.

In analyzing the factors associated with the presence of complications, women with a higher gestational age had more complications in the present study, which is akin to the findings of other studies. 13 Characteristics inherent to the second gestational trimester, such as a higher uterine volume, greater amount of retained products of conception, and possible chorioamnionitis may increase the chance of uterine atony, uterine perforation and sepsis. 14 15

In the present study, ∼ 30% of the patients became pregnant despite using a CM. Although long-acting reversible contraceptives (LARCs) such as intrauterine devices have a lower failure rate, in Latin America, they are still underused compared with other CMs. In Brazil, the predominant CMs used are oral contraceptives, female sterilization, and male condom, possibly due to the high cost, ignorance regarding LARCs, and lack of trained health professionals, both in counseling and insertion techniques. 16 17 Health promotion, sex education, funding, and team training are tools to popularize LARCs and decrease the rate of unplanned pregnancies. 18 In the present study, women who reported CM failure presented with a 3.4-fold higher risk of developing complications. Unfortunately, it was not possible to differentiate between a spontaneous and a voluntary “early pregnancy loss.” Therefore, all abortions that were not induced for legal reasons were classified as “abortion.” Possibly, some of these women may have taken deliberate unsafe actions to induce gestational loss, as previously demonstrated in other studies. 5

The complication-associated factors found in the present study may be correlated. Women using a CM may not associate their symptoms with pregnancy, leading to a late diagnosis of pregnancy due to a delay in seeking medical attention, which negatively influences the outcome of the patient. 5 19 20 21 The reasons for the delay to seek medical care can be as follows: not considering the need for medical care, not knowing where to seek care, shame, fear of criticism, disagreement with the partner and/or family in relation to pregnancy termination, distance from medical services (for example, in rural areas), concerns regarding absenteeism at work, difficulties with transportation, not having a companion, or difficulty in finding a caregiver for their children while seeking care. 5 19 20 21 This delay may increase the risk of full-blown infection and/or hemorrhage, thereby worsening the patients' general state upon hospital admission. Health professionals should emphasize the failure risks of CMs, despite their safety. Moreover, the use of LARCs and dual-method contraception should be encouraged to reduce the number of unplanned pregnancies, in addition to protection against sexually-transmitted diseases.

We acknowledge the limitations of the present study. It was not possible to establish a cause-effect relationship because the study was cross-sectional. During the data collection period, the use of manual vacuum aspiration (MVA), a method of choice for uterine evacuation in pregnancies up to 12 weeks, had not yet been implemented at our institution. A method less invasive than curettage, MVA has a lower failure risk than medical abortion. 22 In the present study, it was not possible to differentiate between cases of spontaneous and provoked abortion. Unsafe abortion is one of the causes of preventable maternal mortality, varying from 8% to 15% of total deaths worldwide. 4 In countries with few abortion restrictions, the use of safe abortion techniques and an appropriate environment results in fewer complications and negative consequences for women's physical and reproductive health. 11 However, in countries with restrictive laws regarding pregnancy termination, there is a higher incidence of unsafe abortion and associated complications, ranging from 2.4/1,000 women in Brazil to 14.6/1,000 in Pakistan. 3 The sociocultural, religious, and political factors that sustain these laws contribute to poorer-quality care and the stigmatization of these women, who continue to risk their lives by performing unsafe abortions, especially in vulnerable populations, such as low-income or non-white populations. 5 Even in Brazil, where pregnancy termination is legal in selected cases, access to these services is still limited to the capital cities and large centers, leaving an enormous portion of the population unassisted. 23 Alarmingly, some Brazilian health professionals are unaware of the documentation required to provide assistance to these women; consequently, these women, who are already in distress, face difficulties in receiving the necessary care. 23

Despite its limitations, we believe that the results obtained in the present study are valid. The data used were obtained after the implementation of an international multicenter surveillance network for the institution of good clinical practices (the MUSA Network) and of a new method to generate knowledge based on the SIP, called EviSIP. 8 9 We observed that, after the implementation of the surveillance network, the institution updated its abortion management protocol to reduce the use of curettage and implement MVA. Strategies that reduce maternal morbidity and unsafe abortion include the promotion of women's rights, professional training, women's empowerment, decriminalization of abortion, sex education, and equitable access to health, which are objectives that are in accordance with the United Nations' Sustainable Development Goals. Despite the decrease in maternal mortality and increased access to contraceptives, the numbers are still alarming, especially in developing countries, proving that we still have a long way to go. 5 16 17 18 23 24 25

## Conclusion

In conclusion, the main complications observed in women who underwent abortion were hemorrhage and infection. Among the variables evaluated, a higher gestational age and CM failure were associated with a higher risk of developing complications. Using this information, it is possible to improve and plan new health practices and policies for the effective identification of women with a higher risk of developing complications and who require more medical attention and monitoring.
